# Examining Trauma Cognitions as a Mechanism of the BRITE Intervention for Female-Identifying Individuals with PTSD Symptoms and Alcohol Misuse

**DOI:** 10.3390/bs15070872

**Published:** 2025-06-26

**Authors:** Elizabeth A. Lehinger, Molly Joseph, Antoine Lebeaut, Scott Graupensperger, Debra Kaysen, Michele A. Bedard-Gilligan

**Affiliations:** 1 Department of Psychiatry and Behavioral Sciences, University of Washington School of Medicine, Seattle, WA 98195, USA; mollykj@uw.edu (M.J.); alebeaut@uw.edu (A.L.); graups@uw.edu (S.G.); mab29@uw.edu (M.A.B.-G.); 2 Department of Psychiatry & Behavioral Sciences, Stanford University, Stanford, CA 94305, USA; dkaysen@stanford.edu

**Keywords:** sexual assault, PTSD, alcohol use, trauma cognitions

## Abstract

Trauma cognitions have been widely supported as a mechanism of change in post-traumatic stress disorder (PTSD) treatment. Less is known about the mediating role of trauma cognitions in early interventions addressing PTSD symptoms and co-occurring conditions such as alcohol misuse. This study was a secondary analysis of data collected as part of a pilot randomized clinical trial of a single session intervention and four coaching calls (BRITE), adapted from Cognitive Processing Therapy for survivors of a sexual assault that occurred in the past 10 weeks. Fifty-seven adult female-identifying individuals with symptoms of PTSD and alcohol misuse randomized to either intervention or symptom monitoring completed the assessments of PTSD severity, alcohol use, and trauma cognitions at intake, post-intervention, and 3-month follow-up. Mixed-effects models showed trauma cognitions improved significantly in the BRITE condition but did not change in the symptom monitoring condition (*b* = −1.53, *p* < 0.001, *B* = −1.05). Mediation analyses indicated that change in total trauma cognitions and self-blame cognitions did not have a significant indirect effect on the association between condition and PTSD symptoms and average drinks on drinking days, and the proportion mediated was small for PTSD symptoms and average drinks. Preliminary findings indicate reductions in negative trauma cognitions for the BRITE condition, but this is likely one of several factors that play a role in changes in PTSD symptoms and alcohol use in the early recovery period following sexual assault.

## 1. Introduction

Sexual assault (SA) is prevalent, impacting approximately 22% of women (Elliott et al., 2004). SA is commonly defined as non-consensual, unwanted sexual contact of any kind. In the week immediately following a SA, around 81% of survivors exhibit symptoms consistent with post-traumatic stress disorder (PTSD), and approximately 75% of survivors meet PTSD symptom criteria one month later (Dworkin et al., 2023). While many survivors experience natural recovery (i.e., without therapeutic intervention) within the first three months (Galatzer-Levy et al., 2018; Shalev et al., 2019), about 42% of survivors continue to meet PTSD criteria at 12 months post-SA (Dworkin et al., 2023). Furthermore, problematic alcohol use following trauma predicts chronic PTSD and the development of alcohol use disorders (AUD) (Kaysen et al., 2011; McFarlane et al., 2009), with about one third of individuals diagnosed with PTSD developing comorbid AUD (Kessler et al., 1995). These associations highlight the critical importance of addressing both PTSD symptoms and alcohol misuse early in the recovery process.

Additionally, trauma-related cognitions have been implicated in prospective studies of trauma exposure as a key indicator of non-recovery (Dunmore et al., 2001; Fairbrother & Rachman, 2006; Kline et al., 2021). Beliefs about self-blame, negative attributions about PTSD symptoms themselves, and the avoidance of trauma-related cues and reminders have all been found to predict a more chronic course of PTSD (Domino et al., 2020; Dunmore et al., 1999, 2001; Shin et al., 2017). Moreover, this pattern has been found with sexual assault survivors, where early self-blame, negative beliefs about others and the world, and negative beliefs about self all predict chronic PTSD following sexual assault (Kline et al., 2021; Peter-Hagene & Ullman, 2018).

Another predictor of a more chronic course of PTSD following sexual assault is alcohol use (Kaysen et al., 2006, 2011). Alcohol use is highly prevalent among survivors of SA (Langdon et al., 2017). This elevated use is concerning as individuals with a history of SA are at a heightened risk for developing hazardous drinking (i.e., a pattern of alcohol use that increases risk for unfavorable health effects; Langdon et al., 2017; Saunders et al., 1993), often as a means of coping with trauma-related distress (Miranda et al., 2002) and emotional regulation difficulties (Messman-Moore et al., 2015). This pattern of alcohol misuse is associated with several adverse outcomes, including increased PTSD symptom severity (Tripp et al., 2020) and a heightened risk of revictimization (Fedina et al., 2018; Lorenz & Ullman, 2016). This functional pattern wherein individuals use alcohol to manage distressing emotional states related to PTSD symptoms, including intrusive thoughts, hyperarousal, and negative affect, perpetuating a cycle of worsening PTSD symptoms and increasing reliance on alcohol, is termed self-medication (Khantzian, 1997) and is supported by longitudinal studies (Hawn et al., 2020). Given these functional relationships, interventions that concurrently target PTSD symptomatology and alcohol-related behavior may be vital for promoting long-term recovery among survivors of SA.

Delivering interventions in the early recovery period following SA exposure may help reduce the severity and duration of PTSD symptoms (Dworkin & Schumacher, 2018) and reduce the risk of substance misuse (Resnick et al., 2007), yet few acute interventions effectively address both concerns. Indeed, early cognitive and exposure-based interventions among trauma-exposed populations have been shown to reduce PTSD symptoms (e.g., Bisson et al., 2019); however, these interventions typically do not target alcohol misuse. Often, interventions such as supportive care or psychological first aid are provided; however, these approaches lack empirical support for facilitating natural recovery and generally do not target specific symptoms or mechanisms associated with the development and maintenance of PTSD (Figueroa et al., 2022). Alcohol use treatments are generally not designed for the acute phase after trauma recovery, with the exception of stepped care interventions delivered in inpatient medical settings, which have incorporated motivational interviewing for patients with positive toxicology screens (Zatzick et al., 2021). Stepped care has demonstrated reductions in drinking, but only small effects on PTSD symptoms (Zatzick et al., 2021). Given that sexual assault survivors are more likely to present at primary care settings or mental health services rather than inpatient medical care (e.g., Amstadter et al., 2008; Vogt et al., 2022), inpatient stepped care approaches are less likely to be utilized by sexual assault survivors.

In brief, light-touch integrated PTSD and alcohol use interventions represent a promising avenue for indicated prevention by providing accessible, low-burden approaches to interrupt the trajectory of psychopathology following SA (Bedard-Gilligan et al., 2020, 2025, unpublished manuscript; Dworkin et al., 2024). For example, a recently developed intervention (Brief Restructuring Intervention following Trauma Exposure; BRITE), which is based on existing Cognitive Therapy approaches for PTSD (Cognitive Processing Therapy, CPT; Resick et al., 2016), is delivered in one session and four coaching calls, and in a small pilot trial, it demonstrated initial feasibility and acceptability, such that all participants completed the intervention and reported high satisfaction and low distress (Bedard-Gilligan et al., 2020). Interventions such as BRITE may be particularly well suited for early post-trauma contexts, where more intensive treatment approaches are potentially less feasible or acceptable. As empirical support for brief interventions grows, examining theorized mechanisms of change underlying their efficacy may enhance their impact and improve their long-term outcomes in survivors of SA.

Cognitions have not only been implicated as key mechanisms in non-recovery but also appear to be a key mechanism in trauma-focused therapies. A recent systematic review found that trauma cognitions were a significant mediator of PTSD symptom change across 14 studies and a variety of treatment approaches including Prolonged Exposure (PE), Dialectical Behavior Therapy + PE (DBT + PE), Cognitive Processing Therapy (CPT), Cognitive Therapy, and CBT for PTSD (Alpert et al., 2023). Although there have been mixed and inconsistent results of studies examining particular types of trauma cognitions as mediators, self-blame cognitions have the most consistent evidence as a treatment mediator among samples of sexual assault survivors (Alpert et al., 2023). However, it is not known if acute cognitively focused interventions—those delivered within the first 3 months following exposure to trauma—act on these same mechanisms. Examining whether changes in trauma cognitions mediate the effect of an early intervention on PTSD severity would further strengthen the evidence that changes in trauma cognitions are a key mechanism of therapeutic change.

Changes in negative trauma cognitions may also underlie changes in trauma-related sequelae such as alcohol misuse. One study found changes in trauma cognitions mediated changes in PTSD severity among individuals with PTSD and co-occurring alcohol use disorders (McLean et al., 2015), indicating that trauma cognitions remain a potential mechanism of change in PTSD treatment among those with co-occurring alcohol use disorders. If alcohol is used to cope with distress in the aftermath of SA, interventions targeting distressing trauma cognitions may also be effective at reducing coping-motivated alcohol use. A recent study found that reductions in trauma cognitions related to the self and the world were associated with fewer heavy drinking days following an integrated treatment for PTSD and alcohol use disorder (Lyons et al., 2023). Reductions in self-blame cognitions, however, were associated with greater heavy drinking days at follow-up. Additional research is needed to understand the potential mechanistic role of change in trauma cognitions on alcohol use among individuals with PTSD.

The current study sought to evaluate whether changes in trauma cognitions mediate subsequent changes in PTSD symptom severity and the average number of standard drinks consumed on drinking days using data from a pilot randomized controlled trial of BRITE, a CPT-based intervention consisting of a single session and four coaching calls. We hypothesized significant reductions in trauma-related cognitions across assessment time points from pre-intervention to post-intervention, and that these reductions would be greater among those who received the intervention compared to those in the symptom monitoring condition. We examined whether pre- to post-intervention changes in trauma cognitions, both total cognitions and self-blame cognitions, would mediate the relationship between condition and PTSD symptoms and average drinks per drinking day at follow-up. We focused on examining clinical significance rather than statistical significance by examining the proportion mediated by changes in trauma cognitions. The self-blame subscale was chosen based on evidence of self-blame cognitions as a treatment mediator among samples of sexual assault survivors (Alpert et al., 2023), and recent evidence indicating reductions in self-blame may have an inverse association with alcohol outcomes (Lyons et al., 2023). The current study expands on prior work by examining the mechanistic role of cognitive change in the early recovery period for both a treatment and a natural recovery group of sexual assault survivors, and by examining the impact of cognitive change on co-occurring alcohol misuse in addition to PTSD symptoms.

## 2. Method

This study is a secondary analysis of a pilot RCT (NCT02808468) comparing a brief intervention (BRITE) to symptom monitoring (Bedard-Gilligan et al., 2020, 2025, unpublished manuscript).

### 2.1. Participants

Participants were female-identifying individuals (i.e., reported their gender identity as women; *n* = 57) who experienced SA within the past 10 weeks. For this study, SA is defined as any non-consensual and distressing sexual activity ranging from forced penetration to unwanted contact, including touching and kissing. Additional inclusion criteria included (1) meeting criteria for at least three of the four DSM-5 (American Psychiatric Association, 2013) PTSD symptom clusters (i.e., re-experiencing, avoidance, alterations in cognitions and mood, hyperarousal), (2) reporting hazardous drinking, defined as at least one incident of heavy episodic drinking (four drinks or more on one occasion) and at least two negative consequences of drinking in the past month, (3) age 18 years or older, (4) English fluency, (5) no planned absences that would interfere with 5 weeks of participation, (6) access to a telephone, and (7) capacity to provide informed consent. Exclusion criteria included current acute suicidality with intent/plan and current psychosis. Participants ranged in age from 18 to 38 years (*M* = 21.63, *SD* = 3.73), were predominately heterosexual (71.9%), White (61.4%), non-Hispanic (87.7%), mostly worked at least part-time (54.4%), mostly reported an annual personal income less than USD 10,000 (71.4%), and mostly had prior treatment histories (66.7%). All but one of the participants were enrolled as a student at a local university.

### 2.2. Procedures

The study procedures were IRB-approved and conducted via telephone and in-person visits. Participants were primarily recruited using university registrar emails, referrals from university and community organizations, online advertisements, and flyers. Interested individuals completed an initial phone screening, and participants deemed potentially eligible then completed a comprehensive in-person intake assessment. The intake assessment included structured interviews administered by a trained and masked evaluator, as well as the completion of self-report measures. Eligibility was determined through consensus during weekly team meetings.

Participants were then randomized without stratification into either the brief cognitive intervention (BRITE; *n* = 28) or symptom monitoring (*n* = 29) conditions using an Excel-generated random assignment. Participants were informed of the condition to which they were assigned. They completed self-report measures at their intervention session or prior to starting symptom monitoring (week 0) and weekly across four weeks of coaching calls/monitoring (weeks 1–4). Week 4 is referred to as post-intervention. Participants then completed a 3-month follow-up assessment with the same evaluator from intake, who was masked to their intervention allocation. Participants were compensated for completing assessments with a possibility of earning up to USD 150 in gift cards if all assessments were completed.

#### Conditions

Intervention: The BRITE intervention, developed based on Cognitive Processing Therapy (CPT; described in detail elsewhere, see Bedard-Gilligan et al., 2020, 2025, unpublished manuscript; Stappenbeck et al., 2015), consisted of one 90 min in-person session and four consecutive weekly coaching calls (15–20 min each). Delivered by trained PhD-level therapists, the intervention provided psychoeducation, cognitive restructuring strategies, and skill-building exercises targeting PTSD symptoms and alcohol use related to the recent SA. Coaching calls reinforced cognitive strategies and skill building including the review of cognitive restructuring worksheets, reviewed trauma symptoms and alcohol use, and provided tailored support. Supplemental resources such as handouts, worksheets, and recordings were provided to reinforce skills learned during sessions.

Symptom Monitoring: Participants randomized to the symptom monitoring condition completed the same weekly online self-report measures as those receiving the BRITE intervention. They had no other scheduled interactions with the research team unless they requested contact or reported urgent concerns.

### 2.3. Measures

#### 2.3.1. Post-Traumatic Stress Severity

The Posttraumatic Stress Symptom Scale-Interview Version for DSM-5 (PSSI; Foa et al., 2016) is a structured interview that demonstrates excellent reliability and validity. Items correspond with the DSM-5 symptoms for PTSD. Symptoms are rated on a 5-point scale of frequency and severity ranging from 0 (*not at all*) to 4 (*6 or more times per week/severe*). PTSD severity is calculated by summing the 20 items, with higher scores indicating greater symptom severity. The PSSI was administered at intake and 3-month follow-up to assess PTSD diagnostic criteria and symptom severity within the prior two weeks, anchored to the recent index SA experience participants described at screening. In the current sample, internal consistency was good at intake (*a* = 0.80) and excellent at follow-up (*a* = 0.90).

#### 2.3.2. Average Drinks Consumed

The Timeline Follow-Back (TLFB; Sobell & Sobell, 1992) is a structured, calendar-based interview with established reliability (Sobell et al., 1988), assessing frequency and quantity of alcohol use. The TLFB was administered at intake and 3-month follow-up and assessed alcohol use over the prior 30 days. At intake, this measure was used to confirm that participants’ alcohol use patterns over the past 30 days included at least one episode of heavy episodic drinking, defined as consuming four or more drinks on one occasion. For the current study, the average number of drinks consumed on drinking days was calculated as the alcohol severity outcome.

#### 2.3.3. Trauma Cognitions

The Posttraumatic Cognitions Inventory (PTCI; Foa et al., 1999) is a 33-item self-report measure of negative post-traumatic cognitions that demonstrates high internal consistency, test–retest reliability, and strong convergent and discriminant validity (Foa et al., 1999). Each item is rated on a 7-point Likert scale ranging from 1 (*totally disagree*) to 7 (*totally agree*). Items include trauma cognitions about the self, “I am a weak person”, the world, “You can never know who will harm you”, and self-blame, “The event happened because of the way I acted”. The PTCI was administered at intake, the intervention session/week 0 for symptom monitoring, and weekly across 4 weeks of coaching calls/monitoring. For the current study, an average was calculated across all items for the variable total cognitions at each time point. An average was also calculated for the self-blame subscale items based on past research supporting the importance of self-blame in recovery (Alpert et al., 2023; Kline et al., 2021). Internal consistency in the current sample was excellent across all time points for total cognitions (*a* = 0.93–0.97) and good to excellent for self-blame cognitions at intake (*a* = 0.82) and post-intervention (*a* = 0.94).

### 2.4. Statistical Analysis

The descriptive information of the study variables, including trauma cognitions, PTSD severity, and average drinks on drinking days, are presented in Table 1. The tests of regression assumptions were conducted for both the mediator and outcome regression models for both PTSD symptoms and average drinks on drinking days outcomes including tests of linearity, normality of residuals, homoscedasticity, and multicollinearity. The examination of plots of residuals and fitted values indicated the linearity assumption was met for the mediator models. Component and residual plots were examined for the outcome models and indicated that each predictor has a linear relationship with the outcome. Residuals appeared to be normally distributed for the mediator and outcome models based on the examination of QQ plots. Homoscedasticity was supported by the examination of plots of residuals and fitted values as well as the Breusch–Pagan test. The multicollinearity assumption was evaluated by examining Variance Inflation Factors, which indicated that multicollinearity was not present. Study variables were assessed for assumptions of normality, and correlations between study variables were examined. Missing values were present in 7.24% of all values. Of this 7.24%, 21.2% of missing values were post-intervention PTCI scores, 39.4% were follow-up PSSI scores, and 39.4% were follow-up TLFB scores. Condition was not a predictor of missingness, and other potential predictors of missingness were examined such as baseline PTSD symptom severity. None were found to be associated with missingness. Data were otherwise determined to be missing at random; therefore, missing values were addressed using multiple imputation by chained equations (i.e., the “mice” package in R; van Buuren & Groothuis-Oudshoorn, 2011). Variables in the imputation models included the following: condition; pre-intervention PSSI, TLFB, and PTCI; post-intervention PTCI; and follow-up PSSI and TLFB scores. A total of 20 imputations were generated, with final estimates derived from pooling across these imputations to account for variability and improve the accuracy of parameter estimation.

As preliminary steps for the focal mediation analyses, we first evaluated changes in total trauma cognitions over time by fitting a two-level random-intercept mixed-effects model where observations were grouped by time point (0 = intake, 1 = intervention/week 0, 2 = week 1, 3 = week 2, 4 = week 3, 5 = week 4). We examined the interaction between time and condition (intervention, symptom monitoring) as predictors of total trauma cognitions. Next, we fit a linear multiple regression model which regressed post-intervention PTCI scores on treatment condition while controlling for pre-intervention PTCI scores to obtain a post-intervention between-group effect size. Then, residualized change scores for total trauma cognitions were calculated by regressing post-intervention trauma cognitions on pre-intervention trauma cognitions. This approach effectively accounts for the variability in post-intervention trauma cognitions that is explained by pre-intervention trauma cognitions, leaving only the remaining variability that can be understood as the change between the two time points (Castro-Schilo & Grimm, 2018).

Mediation models were conducted using the “mediation” package in R (Tingley et al., 2014). Separate models were examined for PTSD symptoms and average drinks on drinking days in the last month at 3-month follow-up, with both models featuring residualized change in total trauma cognitions as the mediator. Residualized change in self-blame cognitions was also examined as the mediator in separate models. Mediation models were conducted separately for each imputed data set, and results were pooled using Rubin’s rules. For each model, a linear regression was first conducted to estimate the effect of condition (treatment vs. symptom monitoring) on trauma cognitions (path a). Then, a second linear regression was conducted to estimate the effect of cognitions and condition on the treatment outcome (paths b and c’). Mediation analyses were then performed using nonparametric bootstrapping (sims = 1000) for each imputed data set. Given the parent study was a pilot trial with a relatively small sample size, mediation results were evaluated based on clinical significance (i.e., effect sizes informing a forthcoming larger trial) in addition to statistical significance. To better interpret the clinical impact of the mediation effect, the proportion mediated was calculated (i.e., indirect effect/total effect) to quantify how much of the total treatment effect was explained by changes in trauma cognitions.

## 3. Results

See Table 1 for correlations between study variables. Overall, trauma cognitions had moderate-to-strong correlations with PTSD symptoms at pre- and post-treatment and weak correlations with average drinks on drinking days at pre- and post-treatment. There were no significant differences in demographic characteristics, endorsement of childhood sexual abuse, baseline PTSD symptom severity, or baseline average drinks on drinking days between the intervention and monitoring groups.

**Figure 1:**
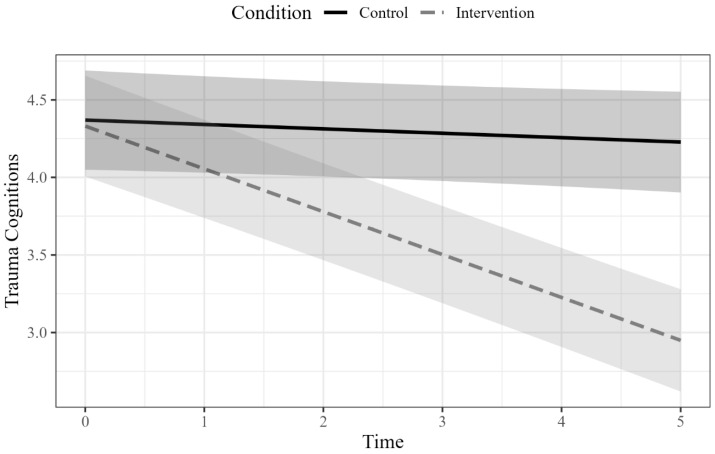
Trauma cognitions over time by condition.

The linear mixed-effects model showed a significant time-by-condition interaction, *b* = −0.25, *SE* = 0.03, *t*(254) = −8.67, *p* < 0.001, indicating that the effect of time on total trauma cognitions differs between conditions. The main effects of time, *b* = −0.03, *SE* = 0.02, *t*(254) = −1.41, *p* = 0.16, and condition, *b* = −0.04, *SE* = 0.23, *t*(65) = −0.17, *p* = 0.87, were not significant. To further examine the significant time by condition interaction, a simple slope analysis was conducted. Results indicated that time was a significant predictor of trauma cognitions in the intervention group, *b* = −0.28, *SE* = 0.02, *t*(254) = −13.63, *p* < 0.001, where trauma cognitions decreased over time. Time was not significantly related to trauma cognitions in the monitoring group, *b* = −0.03, *SE* = 0.02, *t*(254) = −1.41, *p* = 0.16, indicating trauma cognitions did not change over time. Being in the intervention group versus symptom monitoring was associated with a 1.03 *SD* decrease in post-treatment trauma cognitions, after adjusting for baseline. Figure 1 shows the simple slopes of trauma cognitions by condition over time. When looking only at the post-intervention scores, a linear model of pre-intervention PTCI scores and condition predicting post-intervention PTCI scores showed a significant effect of condition (*b* = −1.53, *p* < 0.001, *B* = −1.05). Those in the intervention condition had a greater reduction in trauma cognitions from pre- to post-intervention (*M* = 4.24 to 2.91) compared to those in the symptom monitoring condition (*M* = 4.21 to 4.13).

### 3.1. PTSD Symptom Severity

**Figure 2:**
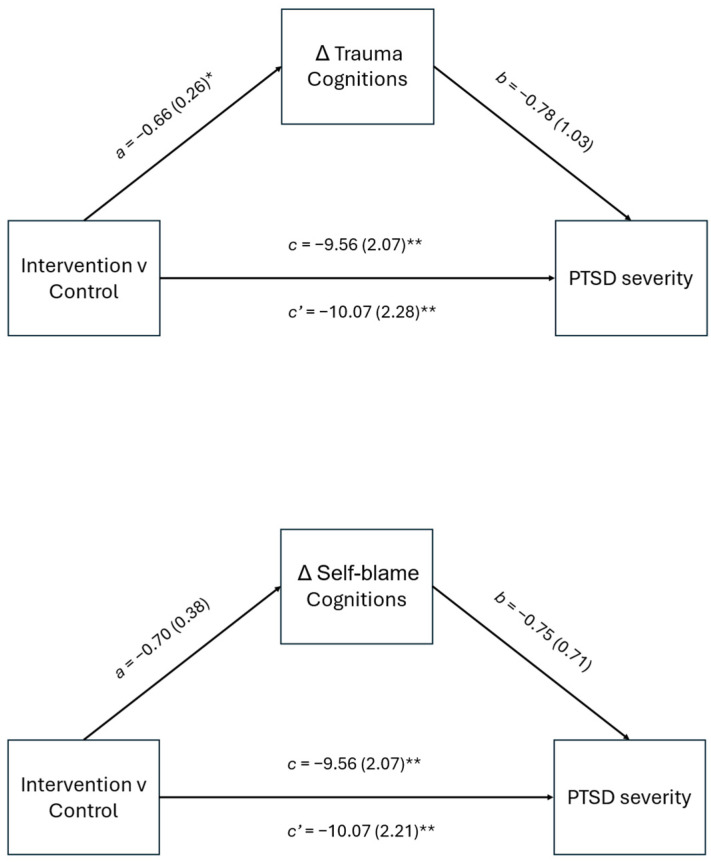
Mediation model of total and direct effects of condition on PSSI scores at three-month follow-up through changes in total trauma and self-blame cognitions. *Note*: unstandardized path coefficients displayed with standard error in parentheses. Partial effects of baseline PSSI score are not depicted in figure. * *p* < 0.05 ** *p* < 0.01.

The results of the mediation analyses with residualized change in total cognitions as the mediator and PTSD symptom severity as the outcome indicated that the total effect of condition on PTSD symptom severity was significant, *b* = −9.56, *p* < 0.001 (See Figure 2). The direct effect of condition on PTSD symptom severity, controlling for trauma cognitions, and pre-intervention PTSD symptom severity was significant, *b* = −10.07, *p* < 0.001. The indirect effect of residualized change in trauma cognitions was not significant, *b* = 0.52, 95% *CI* [−1.38, 2.42]. The proportion of the total effect mediated was 5%, indicating the indirect effect is not clinically significant, and that there may be a suppression effect between residualized change in trauma cognitions and PTSD symptom severity. Suppression may be occurring due to the opposing directions of the indirect and direct effects.

In the model with residualized change in self-blame cognitions as the mediator, the total effect of condition on PTSD symptom severity was significant, *b* = −9.56, *p* < 0.001, and the direct effect was significant, *b* = −10.07, *p* < 0.001. The indirect effect of residualized change in self-blame cognitions was not significant, *b* = 0.52, 95% *CI* [−1.02, 2.07]. Similar to the total cognitions model, the proportion of the total effect mediated was 5%.

### 3.2. Average Drinks on Drinking Days

**Figure 3:**
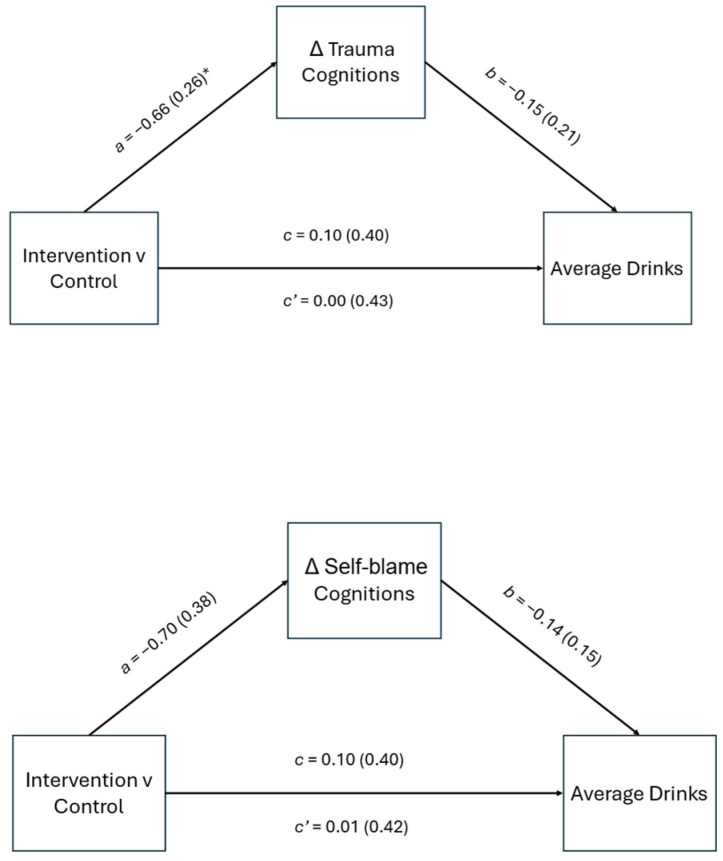
Mediation model of total and direct effects of condition on average drinks on drinking days at three-month follow-up through changes in total trauma and self-blame cognitions. *Note*: unstandardized path coefficients displayed with standard error in parentheses. Partial effects of baseline past-month alcohol use are not depicted in figure. * *p* < 0.05.

In the model with residualized change in total cognitions as the mediator and average drinks on drinking days as the outcome, the total effect of condition on average drinks was not statistically significant, *b* = 0.10, *p* = 0.804 (See Figure 3). The direct effect of condition on average drinks, controlling for trauma cognitions, and pre-intervention alcohol severity was not significant, *b* = 0.00, *p* = 0.996. The indirect effect of treatment condition via residualized change in trauma cognitions was not statistically significant, *b* = 0.09, 95% *CI* [−0.25, 0.45]. The proportion of the total effect mediated was 28%, indicating that reductions in trauma cognitions plays a small role in explaining the effect of condition on average drinks on drinking days. In the model with residualized change in self-blame cognitions as the mediator, the total effect of condition on PTSD symptom severity was significant, *b* = 0.10, *p* = 0.804, and the direct effect was not significant, *b* = 0.01, *p* = 0.988. The indirect effect of residualized change in self-blame cognitions was not significant, *b* = 0.09, 95% *CI* [−0.44, 0.63]. The proportion of the total effect mediated was 23%; however, the proportion mediated for the models with average drinks as the outcome should be interpreted with caution given the total effect is near zero (Table 2).

## 4. Discussion

The current study had two primary goals. First, we examined the effect of a single-session early intervention and four coaching calls for PTSD symptoms and alcohol use after SA on maladaptive trauma cognitions. Second, we examined reduction in trauma cognitions as a mediator of the effect of treatment condition (intervention vs. symptom monitoring) on PTSD symptoms and average drinks consumed on drinking days immediately post-intervention. Total PTCI trauma cognitions and the self-blame subscale items were examined as mediators. As hypothesized, trauma cognitions decreased significantly over time among individuals who received the BRITE intervention, which is a cognitively focused intervention. Trauma cognitions did not significantly change over time among individuals in the symptom monitoring condition. We did not find evidence supporting an indirect effect of changes in total or self-blame trauma cognitions on the effect of condition on PTSD symptoms and average drinks, although a small proportion of the total effect was mediated by changes in cognitions in all models.

The reduction in trauma cognitions in the intervention group indicates the treatment had a large effect on trauma cognitions. At post-intervention, the average response on the PTCI items for the intervention condition was 1.53 lower than that for the symptom monitoring condition on a 1–7 Likert scale. Regarding mechanisms of change, this demonstrates preliminary support for the specificity of action, as the total trauma cognitions did not change in the comparison condition (Carey et al., 2020; Kazdin, 2009, 2011). This finding is compelling considering the degree of change observed from a single-session intervention with four coaching calls. It implies that brief interventions such as BRITE offer a low-burden approach to addressing negative post-trauma cognitions that can be delivered quickly following sexual assault. Given the substantive role that cognitions play in predicting the risk of chronic PTSD, this highlights the potential of a cognitively focused intervention as indicated prevention. As participants repeatedly apply the skills learned to address trauma cognitions, the intervention effects may continue to build over time. Future research should evaluate whether these reductions persist in a larger RCT with a more active control condition and over longer periods of time given the current study only examined pre- to post-intervention change in trauma cognitions.

We did not find evidence of a significant indirect effect of condition on PTSD symptoms through changes in total trauma or self-blame cognitions in this small pilot sample. This could be due to the potential variability among participants in the direction of change in cognitions from pre- to post-intervention, or that a small indirect effect was not detected due to the small sample size. It may also be that cognitions play a critical role in PTSD symptom improvements for some individuals but less so for others. For individuals who have prior trauma histories, if the beliefs were more entrenched, it may be that the intervention was less able to have an effect. Future research should examine prior trauma history and prior PTSD as potential moderators of treatment effects. In addition, the proportion of the total effect mediated was small for PTSD symptoms, suggesting there are likely other factors explaining the effect of intervention on PTSD symptoms. It may be that the intervention engaged other processes known to be beneficial to early recovery following SA such as the social support provided by the therapist or reducing avoidance (Kline et al., 2021; Ullman, 1999).

We also did not find evidence of a significant indirect effect of condition on average drinks on drinking days through changes in total trauma or self-blame cognitions. The PTCI does not measure cognitions specific to self-medication, which may explain why we did not find an association between changes in cognitions and average drinks on drinking days. Furthermore, the direct and total effects in these models were not significant, indicating that there were no significant differences in changes in average drinks between the intervention and symptom monitoring conditions. The BRITE protocol focused on addressing both trauma- and self-medication-related negative cognitions, but this may be insufficient for reducing alcohol use. Participants may have had alcohol motives other than self-medication such as drinking to enhance social interactions or increase pleasure, which may explain the non-significant intervention effects. Although not statistically significant, the negative correlations between self-blame and average drinks in the current study is contrary to our expectations. The work by Lyons et al. (2023) found that reductions in self-blame were associated with greater heavy drinking days following integrated treatment for PTSD and alcohol use. Future studies with larger samples should examine whether reductions in self-blame lead to increases in alcohol use among sexual assault survivors, which may be due to reductions in assimilated beliefs about alcohol causing the sexual assault.

Several limitations of the current study should be noted. Despite being powered to evaluate a time-by-condition interaction, the sample size is relatively small and may be underpowered to detect small indirect effects. Therefore, the non-significant indirect effects should be interpreted with caution. The residualized change score approach has also been critiqued for sensitivity to measurement error and not fully accounting for temporal precedence. Future studies of brief, early interventions following SA should include larger samples and employ more robust statistical approaches for examining trauma cognitions as a mechanism of change preceding changes in PTSD severity and alcohol use, such as multilevel lagged regression or latent change score models. This would allow for an evaluation of the reverse direction of effects, i.e., changes in PTSD symptoms or average drinks predict changes in trauma cognitions. Participants were predominately female-identifying college students, which limits the generalizability of the findings. Future studies should recruit for a diverse representation of sexual assault survivors. We also did not include a measure of alcohol-related cognitions, which would be an important mechanism to test in an integrated intervention. This study also relied on self-report and interview measures that are subject to potential biases such as recall inaccuracies and social desirability. Finally, it is possible that other factors such as the general support provided by therapists in the intervention condition may account for changes in both trauma cognitions and PTSD symptoms. Evaluating changes in trauma cognitions as a mechanism of PTSD and alcohol use reduction may be improved by measuring and controlling for such factors.

The current study found significant reductions in negative trauma cognitions following a brief cognitive intervention delivered in the early recovery period after a recent SA. Reductions in trauma cognitions did not mediate the effect of the intervention on PTSD symptoms and average drinks on drinking days at follow-up, indicating other factors help to explain reductions in PTSD symptoms and alcohol use. Overall, the findings support the use of brief interventions to target negative trauma cognitions, which are a clinically meaningful and malleable treatment target regardless of their short-term impact on PTSD symptoms and alcohol-related outcomes.

## Figures and Tables

**Table 1:** Correlations between study variables.

	1	2	3	4	5	6	7	8
1. Pre PTCI total	-							
2. Post PTCI total	0.48 **	-						
3. Pre Self-blame	0.73 **	0.33 *						
4. Post Self-blame	0.46 **	0.86 **	0.50 **					
5. Pre PSSI	0.52 **	0.33 *	0.28 *	0.20	-			
6. Follow-up PSSI	0.50 **	0.55 **	0.25	0.51 **	0.60 **	-		
7. Pre Average Drinks	0.01	0.02	−0.22	−0.14	0.18	0.16	-	
8. Follow-up Average Drinks	−0.25	−0.11	−0.29	−0.10	−0.23	−0.20	0.44 **	-
*M*	4.22	3.51	4.82	3.95	32.84	16.75	4.1	3.19
*SD*	0.85	1.16	1.24	1.67	8.33	10.3	1.45	1.60
Range	2.15–6.15	1.09–5.73	1.8–7	1–6.6	11–54	3–48	1.86–7.89	0–7.30

*Note*. PTCI = Posttraumatic Cognitions Inventory. PSSI = Posttraumatic Stress Symptom Scale-Interview. * *p* < 0.05, ** *p* < 0.01.

**Table 2:** Summary of mediation analysis.

Outcome	*b*	*SE*	95% CI (Lower, Upper)	*p*
PTSD symptom severity				
**Total trauma cognitions as mediator**				
Condition → Δ Trauma Cognitions (a path)	−0.66	0.26		0.014
Δ Trauma Cognitions → PTSD symptom severity (b path)	−0.78	1.03		0.461
Condition → PTSD symptom severity (total effect, c path)	−9.56	2.07		<0.001
Condition → PTSD symptom severity (direct effect, c’ path)	−10.07	2.28		<0.001
Indirect effect (a × b)	0.52	0.98	[−1.39, 2.44]	
**Self-blame cognitions as mediator**				
Condition → Δ Self-blame Cognitions (a path)	−0.70	0.38		0.071
Δ Self-blame Cognitions → PTSD symptom severity (b path)	−0.75	0.71		0.303
Condition → PTSD symptom severity (total effect, c path)	−9.56	2.07		<0.001
Condition → PTSD symptom severity (direct effect, c’ path)	−10.07	2.21		<0.001
Indirect effect (a × b)	0.52	0.79	[−1.02, 2.07]	
Average drinks on drinking days				
**Total trauma cognitions as mediator**				
Condition → Δ Trauma Cognitions (a path)	−0.66	0.26		0.014
Δ Trauma Cognitions → Average drinks (b path)	−0.15	0.21		0.498
Condition → Average drinks (total effect, c path)	0.10	0.40		0.804
Condition → Average drinks (direct effect, c’ path)	0.00	0.43		0.996
Indirect effect (a × b)	0.10	0.18	[−0.25, 0.45]	
**Self-blame cognitions as mediator**				
Condition → Δ Self-blame Cognitions (a path)	−0.70	0.38		0.071
Δ Self-blame Cognitions → Average drinks (b path)	−0.14	0.15		0.361
Condition → Average drinks (total effect, c path)	0.10	0.40		0.804
Condition → Average drinks (direct effect, c’ path)	0.01	0.42		0.988
Indirect effect (a × b)	0.09	0.27	[−0.44, 0.63]	

## Data Availability

The data presented in this study are available on request from the corresponding author due to ethical restrictions.
